# Informal Caregiver Stroke Program in Geriatric Rehabilitation of Stroke Patients: A Qualitative Study

**DOI:** 10.3390/jcm12093085

**Published:** 2023-04-24

**Authors:** Mariska Vielvoye, Christa S. Nanninga, Wilco P. Achterberg, Monique A. A. Caljouw

**Affiliations:** 1 Zorgwaard, 3262 HW Oud-Beijerland, The Netherlands; 2 Zonnehuisgroep Vlaardingen, 3136 EA Vlaardingen, The Netherlands; 3 University Network for the Care Sector South Holland, Leiden University Medical Center, 2333 ZA Leiden, The Netherlands; 4 Department of Public Health and Primary Care, Leiden University Medical Center, 2333 ZA Leiden, The Netherlands

**Keywords:** informal caregiver, geriatric rehabilitation, stroke, caregiver burden, qualitative

## Abstract

This study aimed to understand and gain insight into an informal caregiver program for caregivers of older stroke patients, which incorporates both skills training and education, in relation to caregiver burden. Semi-structured, in-depth interviews with individual informal caregivers were conducted at admission, at discharge, and six weeks after discharge. The program consisted of informal caregiver meetings, walk-in days, psychoeducation, and weekend leave after admission to a rehabilitation stroke unit in a nursing home. Eleven informal caregivers participated in the study. The informal caregiver meetings and walk-in days provided more insight into the (level of) functioning of the stroke patients, more skills in guiding them, and better communication with the multidisciplinary care team. During weekend leave, caregivers experienced what their roles as caregivers entailed. Six weeks after discharge, caregivers said that they did not miss any guidance during admission and that they were positive about the future. About half of the caregivers found the caregiver situation disappointing, and combining care tasks with daily tasks appeared to be difficult. Offering informal caregivers a tailor-made program during rehabilitation and good communication helps to diminish caregiver burden in the post-discharge phase when their relatives are back home.

## 1. Introduction

Every year around 43,000 people in the Netherlands have a stroke, also known as a cerebrovascular accident (CVA) [1]. Stroke is one of the main causes of mortality, chronic and severe disability, and reduced quality of life worldwide [2]. A large proportion of older stroke patients, who are 65 years old and older, return to their homes, but many people have permanent limitations in daily activities due to physical, cognitive, emotional, and/or behavioral problems [3]. Having an informal caregiver appears to be an important determining factor for returning home [4]. Although professional help is offered at home, most care is provided by informal caregivers, including family members [5]. Among family members who care for individuals with stroke, the primary caregivers are often spouses and children [6]. Their caring roles typically involve providing assistance with daily activities, including physical care, and provision of emotional support [7].

As a result of the sudden onset of stroke and continuous uncertainty, caregivers face increasing adverse effects from their new caregiving roles and responsibilities, not only for themselves but also for older stroke patients [8,9,10]. This effect is referred to as caregiver burden, and these adverse effects are defined as “the extent to which caregivers perceive the adverse effect that caregiving has on their emotional, social, financial, and physical functioning” [11]. This caregiver burden can lead to an increased level of stress, anxiety, mood problems, reduced health status, reduced quality of life, fewer social contacts, and reduced well-being [8,12]. The risk of overburdening the informal caregiver seems to increase as the stroke-related physical and cognitive problems of the patient become more severe [8]. Moreover, patients are discharged home after rehabilitation faster than in the past, possibly resulting in a higher burden on caregivers.

Informal caregivers’ needs are often given low priority in the management of stroke rehabilitation [13], which is a problem because lack of education of informal caregivers about the stroke recovery process is known to be associated with increased anxiety, stress, fear, and uncertainty about the future [14]. Anxiety and depression are common in caregivers of older stroke patients and are closely related to care burden. Appropriate home care guidance, psychological counseling, and social support should be provided to caregivers to reduce their physical and mental burden [15]. Family caregivers of older stroke patients experience challenging difficulties, such as a lack of support and the practical knowledge and skills to care for persons with stroke at home. These aspects also negatively influence the caregivers’ burden and quality of life, their utilization of health services, and hospital readmissions of older stroke patients [16]. Moreover, poorly prepared caregivers present a safety risk for patients and caregivers and may increase preventable readmissions [17]. Conversely, educating caregivers about the management of stroke improves health outcomes [17,18]. Providing stroke education may be one way to eliminate caregiver feelings of uncertainty post-stroke [17]. Patient care education reduces the burden of care and improves the quality of life of the caregivers of older stroke patients [19]. Training caregivers in skills for the day-to-day management of older stroke patients during rehabilitation reduces caregiver burden [20]. Conversely, addressing these needs is beneficial and is supported by evidence from a scoping review that showed the importance of caregivers being informed and educated about various aspects of the disease process, recovery, and interventions [21].

This study aimed to understand and gain insight into an informal caregiver program, which incorporates both skills training and education, in relation to caregiver burden.

## 2. Materials and Methods

In this qualitative study, data were collected by means of semi-structured, in-depth interviews with individual informal caregivers of older stroke patients admitted to a geriatric rehabilitation center. The interviews were repeated with the same participants at three different time points (in the first two weeks of admission, in the discharge phase, and six weeks after discharge). The rationale for repeated interviews was to explore the course of caregiver burden. The chosen moments (after admission, prior to discharge, and after discharge) were important moments during and after geriatric rehabilitation. This approach allowed us to describe what the added value of the caregiver program was with respect to caregiver burden. The first and second interviews were conducted in the geriatric rehabilitation center, and the third interview was conducted online.

### 2.1. Participants

Informal caregivers of older stroke patients, who are admitted to the stroke unit of Zonnehuisgroep Vlaardingen in the Netherlands, were eligible to participate in this study. They were recruited between June and August 2019. The research was conducted before the COVID pandemic. The inclusion criteria were: (1) the informal caregivers must be motivated for training; (2) the rehabilitation team expects the patient will be discharged home; and (3) the caregiver has adequate proficiency in the Dutch language.

After admission, all informal caregivers of older stroke patients were invited to participate. They received an information leaflet and were subsequently contacted by telephone. All participants participated in this study voluntarily and gave written informed consent.

### 2.2. Setting

The stroke unit is located in a geriatric rehabilitation center in a nursing home. Older stroke patients were admitted for multidisciplinary treatment to this unit after discharge from regional acute stroke units. The multidisciplinary team consists of elderly care physicians, physical therapists, occupational therapists, nutritionists, speech therapists, movement therapists, psychologists, social workers, and nurses. This multidisciplinary team specializes in stroke rehabilitation.

### 2.3. Informal Caregiver Stroke Program

The intervention evaluated in this study, named “the informal caregiver stroke program,” was developed with practical knowledge provided by therapists and informal caregivers. All participating informal caregivers had an introduction meeting with a social worker. They were subsequently asked to participate in the intervention, which consisted of four parts: (1) three informal caregiver meetings; (2) walk-in days to observe the therapy given by the physiotherapist, occupational therapist, speech therapist, and psychologist; (3) weekend leave if possible; and (4) psychoeducation in case of significant cognitive impairments in the older stroke patient if indicated. The program was tailor-made, and therefore, it was not mandatory to participate in all parts.

#### 2.3.1. Informal Caregiver Meetings

Three informal caregiver meetings were offered in groups to informal caregivers, each with a different theme, namely “The visible and invisible consequences of a stroke”, “dealing with a loved one with a stroke” and “trajectory after clinical rehabilitation”. These meetings were offered every two weeks on a different day by a psychologist and social worker.

In the first meeting about the consequences of a stroke, participants were informed about what a stroke means for the patient, changes in thinking, behavioral and character changes, emotional problems, and fatigue. For most participants, these problems are the least visible stroke-related problems that are difficult to understand and hard to manage. The second meeting addressed the different phases in stroke rehabilitation, the mourning process and ways to support it, and the changed caregiver role. In the third meeting, participants were informed about the discharge phase, financial consequences, and aftercare possibilities, such as home care and other organizations that can offer support. During the meetings, participants were given the opportunity to ask questions and exchange experiences.

#### 2.3.2. Walk-In Days

After a two-week diagnostic phase, informal caregivers were individually invited by the occupational therapist to observe the therapy. Informal caregivers were called and invited, and this step was organized by individuals. During the walk-in days, informal caregivers received skills training and information about the level of functioning of the older stroke patient. A maximum of two informal caregivers could observe during the walk-in days.

#### 2.3.3. Weekend Leave

Weekend leave was offered to let the older stroke patients and caregivers experience what it is like to be at home. It involved going home for a day or part of a day under the guidance of the informal caregiver, to see whether the patient is able to move around the house safely and perform various daily activities. A leave was planned as soon as:

- The house was suitable for safe entrance and passage to the toilet and living room;
- The informal caregiver was sufficiently skilled by the occupational therapist to guide and observe the patient;
- The patient was able to move safely (possibly under supervision);
- The patient was medically stable.

The duration of the weekend leave depended on various factors, such as the degree of independence, the ability of the patient and caregiver to manage and persevere the weekend leave, and the suitability of the home. The duration of the weekend leave was determined in the multidisciplinary team meeting. If the patient was dependent on home care, weekend leave would last a maximum of one day.

The weekend leave was self-evaluated by the informal caregiver and afterward evaluated by the occupational therapist. The patient received an evaluation form with the goals of the weekend leave and a number of questions. Any new goals that arose as a result of the evaluation were discussed in the multidisciplinary meeting.

#### 2.3.4. Psychoeducation

Every patient who was admitted to the stroke unit was screened by a psychologist. Only if there were cognitive problems were the results explained to the informal caregiver and the patient, which is a customized process. Cognitive problems may affect the relationship with the caregiver. Therefore, changes in the roles, habits, and behaviors of the patient were explained during psychoeducation. The psychologist discussed how to manage these changes in daily life with the informal caregiver.

### 2.4. Data Collection

Data were collected until data saturation was achieved through semi-structured, in-depth interviews with informal caregivers.

The burden of informal caregivers was discussed based on three phases in the rehabilitation process: the first two weeks of admission, the discharge phase, and six weeks after discharge.

In the first interview, data were collected about the experiences of being a caregiver, experiences with the multidisciplinary team, perceived burden, and expectations about discharge and the future. The same questions were asked in the second and third interview. Questions about information meetings, joining and observing the therapy, and weekend leave were added in the second interview. The third interview focused on experiences with being at home and the question of whether caregivers were sufficiently prepared for the patient returning home.

The patient’s level of independence was recorded two weeks after admission by means of the KATZ. The KATZ-ADL scores the degree of independence. The six questions address the patient’s ability to bathe, dress, visit the toilet, and make transfers and his or her continence and feeding. For each activity, the patient is able to perform without supervision or personal assistance, the older stroke patient receives one point. Summed scores range from 0 to 6, with higher scores indicating a higher degree of independence. The KATZ-ADL was scored by the informal caregiver [22,23].

### 2.5. Data Processing and Analysis

The interviews were digitally recorded, files were transcribed verbatim and coded. Thematic analysis was used, and coding and analysis were performed by the first and second authors and discussed with the last author. The discussion served an analytic purpose, to critique discrepancies and to reach agreement on the codes.

Anonymity and confidentiality were guaranteed, and any names of people appearing in the quotes were deleted from the written texts or anonymized. A combination of inductive and deductive coding was applied. First, we used inductive coding to create codes based on the qualitative data, to look for themes raised by the informal caregivers themselves. Subsequently, we compiled a code list by means of deductive coding, which included the themes in which we were interested to evaluate the training. A code list was then prepared and used to code the interviews (see Table 1).

## 3. Results

### 3.1. Study Population

Forty-seven informal caregivers were invited to participate in this study, 18 of whom consented to participate. Reasons given for not participating in the study were informal caregivers were too busy with daily activities or work; some older stroke patients were discharged within two weeks after admission, and most informal caregivers were busy organizing everything before the discharge.

Between May and October 2019, 18 informal caregivers of older stroke patients were followed. Of these 18 informal caregivers, seven stopped early due to discharge within two weeks after admission, hospitalization, or death of the older stroke patient. These dropouts resulted in a final study population of 11 participants for evaluation. The characteristics of the study population (*n* = 11) are reported in Table 2. The age of the informal caregivers ranged from 31 to 78 years old, 82% were female, and about 64% were spousal informal caregivers. The older stroke patients’ age ranged from 68 to 88 years old, and 18% were female.

The overall independence of the older stroke patients was high, particularly higher than 4 on the KATZ. The stroke patients were highly dependent on the categories of washing, dressing, toileting, mobility, food, and continence. One older stroke patient had a low independence score on the KATZ.

### 3.2. First Two Weeks of Admission

#### 3.2.1. Knowledge, Skills and Communication

Many caregivers experienced problems with the provision of information. A few caregivers said they felt anxious and were suspicious because of inadequate communication about the functioning of the older stroke patient. In addition, caregivers had difficulty letting go of the care for their loved one.

Participant 16 at admission: “… Yes, that is something that concerns me; especially in the beginning, it bothered me because I felt things weren’t organized adequately. Is she drinking? Is she eating? Does everyone realize she can’t see? That was really difficult. Kept me up at night.”

During admission, more than half of the informal caregivers said that they dreaded the discharge of their loved one. Caregivers felt unsure about their own knowledge of stroke, and almost all participants considered themselves insufficiently skilled in guiding their loved ones.

Participant 16: “We were told some things by the doctor at the hospital, but no, that’s really all I know.”

Participant 15: “Well, as long as he is here, I want to be there as much as possible so that I can learn, including how to communicate, because if he can never speak properly again, then I should be able to learn, shouldn’t I?”

During admission, most of the informal caregivers said they were dissatisfied with the communication with the multidisciplinary team. Most informal caregivers did not know which person to turn to when they had questions about their loved one and what the best time was to call. Many informal caregivers missed a single point of contact and went home not feeling confident about their partner being in good hands.

Participant 16 at admission: “No, I wasn’t informed, and I would like to be, to have a contact person I can approach with my questions at other times.”

#### 3.2.2. Informal Caregiver Burden

Many informal caregivers indicated in the interviews that they had little energy and that caring for their loved ones was too stressful. Some caregivers considered it a duty to visit their partners. Due to the energy and time that these visits required, the caregivers found it difficult to combine this task with other (care) tasks and obligations.

Participant 11 at admission: “Even when I have no energy, I still have to find it somewhere.”

Participant 17 at admission: “No, I am not getting anything done. The first week, I didn’t sleep and was very tired. It is hitting home now… I have to find a balance between here and home, to keep myself going… Yes, I still have to do all that, a little shopping, but that will be OK. But this week, I was still so tired; then I am happy to be home and, for a while… I mainly feel it’s my duty, but I also really want to be here.”

Caregivers explained that they felt able to perform care tasks for a short time but did not have sufficient energy to continue doing so in the long term. At the admission of their relative to the rehabilitation stroke unit, slightly less than half of the caregivers indicated having insufficient energy to care for their loved one.

Participant 16 at admission: “Yes, I don’t know if I have enough. I do need enough energy. It’s not easy. It means putting my own life and social activities on the back burner. You can do that for a while, like I did last year. I am looking forward to getting back to calmer waters and having a little more room to breathe.”

Caregivers were not only concerned with care tasks, but these care tasks were also performed at the expense of their own self-care.

Participant 3 at admission: “Well, recently a friend asked me, ‘How are you doing?’ I said, ‘So far, you’re the only one who has asked about me.’ Everyone asks how dad is and how terrible and all that. That’s OK, but… well… And that can make me angry sometimes. Well, not angry, but it makes me think hmm….”

It is often frustrating for caregivers that all attention is focused on the person who has been admitted to the geriatric rehabilitation center and not on them, while they also need attention and care. They also need to share their feelings of frustration, anger, sadness, guilt, or anxiousness. Caregivers experienced difficulty asking for help and did not talk enough about their emotions and feelings. Almost all respondents worried greatly, especially at night, which made them feel tired.

Participant 17 at admission: “Yes, I wake up three to four times… Yes, what also happens in the beginning, something comes into your mind, and then you worry; you shouldn’t, but you do.”

In the first two weeks of admission, informal caregivers need good communication from the multidisciplinary team. Clarity about the goals that need to be achieved before the patient can return home and the expected discharge date is essential for caregivers to be able to prepare. Adjusting the target date without explanation to a short-term discharge causes substantial stress. Informal caregivers need clear communication about the patient’s level of independence and one person to maintain regular contact. Many informal caregivers experienced burdens during this phase. They were tired and had low energy levels. They were uneasy about the upcoming discharge and lacked adequate knowledge about stroke and their own skills to properly guide and care for their loved one. The informal caregivers worried greatly and had difficulty asking for help.

### 3.3. Discharge Phase

#### 3.3.1. Communication

In the discharge phase, more than half of the informal caregivers were positive about communication with the care team, and most of the informal caregivers were positive about the communication with the treatment team. Informal caregivers need feedback from the multidisciplinary meeting. They want to know about the progress and possible stagnation in the recovery of their loved one, and they want to be involved in the rehabilitation process.

Participant 12 at discharge: “I would also like to know what the results are now… is it the same conclusions every week, but they could also be different, or the effects can be different, but that is what we want—as family, I want to know about the progress and the other things.”

Almost all caregivers indicated that communication improved during admission. The walk-in day meant that informal caregivers had a better understanding of which disciplines and faces were involved and to whom they could turn with questions.

Participant 3 upon discharge: “Yes, to get more information and an answer about who the involved parties are. Then, you no longer have to search and approach everyone.”

#### 3.3.2. Informal Caregiver Burden

A few weeks before discharge, informal caregivers experienced a peak in the burden. Many things had to be organized before discharge, and only after everything was arranged could caregivers relax. The interviews with informal caregivers showed that, when several informal caregivers were involved, care tasks and visits to the care recipient could be divided, and they felt less overburdened.

Participant 10: “We also need to be clear to his environment and also towards my father that he should also ask his neighbor or his girlfriend or sister for help. We can’t always be the only ones because that is just too much. We’ll have to see how that goes. I hope it will work itself out.”

The relationship to the person who needed help also determined the degree of overload. Generally, children dreaded the discharge more than partners. Particularly at discharge, children of older stroke patients seemed to worry more than partners.

Participant 11: “Yes, it is a lot of organizing; home care is coming tonight, but I know him. I’ll also have to organize keys to the house because if he doesn’t answer the door… so I have to go and talk to the housing association. He has to be there as well because of the General Data Protection Regulation.”

Most informal caregivers were happy at discharge because their loved ones were able to return to their own familiar environment. The informal care situation had improved compared to the admission, and informal caregivers experienced caring for their loved ones not as burdensome.

#### 3.3.3. Informal Caregiver Stroke Program

At discharge, most caregivers thought they had obtained enough knowledge about the consequences of a stroke. Almost all participants had participated in the informal caregiver meetings or psychoeducation. Caregivers felt the informal caregiver meetings were a pleasant way to obtain stroke-related information, and as a result, they were better able to understand their loved one.

Participant 4: “You understand things you did not understand before.”

The provided written information following the informal caregiver meeting was also experienced as positive. Contact with peers felt fine for some informal caregivers, but opinions about the exchange of experiences were mixed. Some caregivers indicated that exchanging experiences with other informal caregivers was burdensome and they preferred to discuss their own problems in one-on-one contacts with a social worker.

Participant 14: “The information was good; sharing experiences was too much.”

It was remarkable that partners in particular went to the informal caregiver meetings. Partners indicated that they often visited their loved one daily, so they were still at the clinic when the meeting took place. In addition, older partners were often less skilled in searching for information online. Other informal caregivers, e.g., children, often experienced difficulty combining their jobs and visiting the informal caregiver meetings.

Almost all informal caregivers who were positive about the communication with the treatment team had participated in the walk-in days. Thanks to walk-in days and informal caregiver meetings, informal caregivers were better able to assess the actual functioning of their loved one. Informal caregivers indicated that walk-in days contributed to their skill level, which increased their self-confidence.

Participant 10: “Yes, I thought the observation days were really nice anyway. The disadvantage for my father is that I am no longer able to blindly accept everything he says because his memory is affected. So sometimes when he said something, I thought: Yeah right… you walk with a walker, but all I see is you sitting in a wheelchair… So when I joined the therapy, I could see what he was able to do…. So I thought it was very helpful to see; also, so you know you have to walk on his left side, make sure he doesn’t drag his leg, because then you have to have a rest and so on. I kind of learned how I could take him for a walk.”

Almost all caregivers who attended the walk-in day felt they had sufficient skills to guide their loved one. Psychoeducation, provided to caregivers of patients with significant cognitive problems, had been experienced as positive.

Participant 10: “Yes really good; she is very clear and very honest. She said: As it stands now, this is really dementia, and he really is not well enough to drive a car, and very concrete things like that, that it was just clear what he is and isn’t capable of and how he is now. That was really good.”

Caregivers said that the weekend leave gave a clear picture of how things would be after discharge. By means of the weekend leave, caregivers experienced what their role as caregiver entailed and what guidance was expected from them.

Participant 9 after weekend leave: “Yes, the weekend leave was positive because it made both my mother and father more confident about the discharge. But it did mean that, both Thursday and Friday, I was completely occupied with his leave and that he is coming home. And arranging incontinence material, physio, speech therapy, and adjustments.”

Participant 3 at discharge about weekend leave: “Yes, how to deal with it better and, uh, in the beginning, I thought, ‘How does that work?’ But he’s living here, and he just does his thing.”

### 3.4. Six Weeks after Discharge

Informal caregivers did not miss guidance during admission. Most of the informal caregivers did an average of one walk-in day, and they recommended offering more walk-in days. Almost all respondents were positive about the future. The perceived burden of care was not very high, and most of the caregivers were happy.

About half of the caregivers found the discharge disappointing because it proved difficult to combine care tasks with daily tasks. More than half of the informal caregivers experienced that they were overburdened six weeks after discharge. Some informal caregivers indicated that the transfer between the rehabilitation stroke unit and the institutions at home, such as home care and the pharmacy, was not organized adequately.

Participant 10: “No, that was disappointing, also because you… this was a completely independent man, and now everything, from paying bills, he dropped his phone, so now he needs a new one… Just everything you take for granted. Look, my dad can’t just go to the store and buy new pants, you know. He needs help with everything, which I never realized because he was independent. That was disappointing. There is so much coming at you, administrative, financial, practical.”

Participant 11: “And no further instructions agreed about catheter care, home care was not informed about this, which I found odd, because he went home with a catheter. Apparently, there was no handover to home care that home care should deal with the catheter.”

## 4. Discussion

In this qualitative study, informal caregivers of stroke survivors discussed their experiences with the informal caregiver stroke program. Involving caregivers intensively during clinical rehabilitation by means of offering education and skills training to the caregivers could be beneficial in diminishing caregiver burden. The informal caregiver stroke program differs from all existing interventions for informal caregivers of older stroke patients because of its focus on information, education, and skills training in both the clinical setting and the home environment. It is clear that interventions with only a psychoeducational focus are not as effective and might even result in poor social and satisfaction outcomes [24]. Studies that incorporate skill building (hands-on caregiver training) with psychoeducational strategies, as was the case in our study, tend to be much more effective at improving caregiver and survivor outcomes than psychoeducation alone [18,24,25]. As far as we know, no research has been performed on understanding and gaining insight into an informal caregiver program, which incorporates both skills training and education, in relation to the caregiver burden.

Informal caregivers were offered a total of five sessions, namely three informal caregiver meetings, at least one walk-in day, and if indicated, one psychoeducation meeting. In addition, there was at least one weekend leave. The goals of the intervention were largely aimed at preparing the caregivers for the home situation, where the frequency was not perceived as overburdening [21]. The caregiver stroke program was offered tailor-made [26], providing a more inclusive environment that better supports and prepares caregivers for their new role [27].

The interviews revealed different experiences and needs in the different stroke rehabilitation phases. The needs relevant for reducing burden were linked to communication, knowledge, and skills.

### 4.1. Communication

During inpatient stays, the communication of the multidisciplinary team was initially experienced as poor. Caregivers actually navigated a new/unknown environment without clear communication [27]. The walk-in day appeared to be an important intervention to increase satisfaction with communication. As a result, caregivers experienced a gradual improvement in communication with the multidisciplinary team. Communication served to reassure caregivers that they could go home knowing their partner was in safe hands. However, in cases of clear communication by the multidisciplinary team from the beginning, caregiver burden was expected to be less. As a consequence, confidence in the treatment team should increase. Caregivers would then entrust their partner to the care team and go back home after a visit with their mind at ease, having more time for themselves.

### 4.2. Knowledge

In this study, two different information sessions were offered to the informal caregivers, i.e., informal caregiver meetings and, in case of cognitive problems in the older stroke patient, an individual appointment for psychoeducation. In the discharge phase, uncertainty among the informal caregivers who attended informal caregiver meetings about their knowledge level diminished, which was also mentioned in other studies [18,28,29].

The informal caregivers gained knowledge about the clinical aspects of stroke, prevention, treatment, and functional recovery, which were information needs revealed by other studies [30]. Informal caregivers liked receiving information and were pleased about the opportunity to read it again in the handouts that were provided. The informal caregiver meetings helped informal caregivers to prepare for and adjust to the new situation [28]. Informal caregivers felt better able to explain the older stroke patient’s behavior. Psychoeducation, also recommended in other studies [24], also contributed considerably. Information-giving interventions improved caregiver knowledge for stroke caregivers [31]. Spouses in particular visited/attended the informal caregivers’ meetings.

Children of older stroke patients were often more proactive and actively searched for information on the internet, among other things. In addition, children are less available to participate in informal caregiver stroke programs due to their daily activities and work. Offering video material can meet the needs of the children of older stroke patients, and the delivery of interventions via telephone and via the Web may be beneficial approaches [24]. The disadvantage is that informal caregivers find incorrect information on the internet. It is important to guide informal caregivers in the search for information by, for example, listing reliable websites [26,30].

### 4.3. Skills

Skills training was provided during walk-in days and the weekend leave. The walk-in days addressed moving and lifting, exercises, and psychological changes [30]. Training caregivers in basic skills of moving and handling and facilitation of activities of daily living reduces burden of care [23]. Caregivers who attended walk-in days felt more confident about their skills, which has also been mentioned in other studies [18,29]. In addition, the walk-in day increases the insight of the informal caregivers into the functioning of the older stroke patient. Caregivers liked the individualized training, which was tailored to their situation, offered during the walk-in days [29,30]. Planning a walk-in day proved to be time-consuming due to the limited availability of informal caregivers and occupational therapists.

The weekend leave increases self-confidence surrounding the discharge. It is necessary to plan the weekend leave at the right time in the rehabilitation process, carefully considering the readiness of the stroke survivor and caregiver. The experience of the weekend leave provides patients and caregivers with insight into life after stroke and can inform therapy and help to prepare patients and families for the transition home [32].

A weekend leave is only possible if the home is sufficiently accessible, and the older stroke patient and informal caregiver are sufficiently taxable. Scheduling a weekend leave too close to the discharge date can cause a spike in the informal caregiver burden. The pressure on informal caregivers increases, especially when adjustments have to be made to the house or when the new situation is very different from the situation before the rehabilitation process. It is important that informal caregivers be given enough time between weekend leave and discharge.

### 4.4. Post-Discharge Needs

Six weeks after discharge, informal caregivers indicated not missing supervision. Almost all informal caregivers considered themselves capable enough to properly guide the older stroke patient and were positive about the future. Caregivers find it difficult to combine care tasks with daily activities. It is therefore important to look carefully at the amount of guidance to be offered. It must be feasible for busy caregivers [24]. Informal caregivers indicated that partner roles have changed and that they are hopeful about the future. Informal caregivers often expect it to be the way it was before the stroke, and they realized that this expectation was not true. Follow-up counseling at home is essential to prevent additional burden on the informal caregiver.

Most studies measured only short-term outcomes. Long-term outcomes should be assessed to find new ways of enhancing existing interventions and using boosters to achieve more positive outcomes in the long term.

### 4.5. Strengths and Limitations

This qualitative study adds significantly to our understanding of informal caregivers’ experiences and burden [33]. Although the sample size in this study was relatively small, data saturation or information power was shown after interviewing 11 participants, and as a result, it was not necessary to recruit new participants [34]. The interviews that we conducted contain much rich information; therefore, there is adequate and sufficient information power to develop new knowledge [34]. Informal caregivers who were overtaxed on admission found participation in this investigation too burdensome, which may mean that taxation problems may be greater than shown in this study.

It might have been helpful to learn more specifically how children can be involved more intensively in the rehabilitation process because their work and caring for their children resulted in their not being able to come to the rehabilitation center as often as they might have wanted. Using video calls or online informative videos can inform children remotely.

## 5. Conclusions

This study is important for informal caregivers, who are often forgotten during the rehabilitation process. It is necessary to guide caregivers so that an optimal home situation is created for both the caregiver and the patient. Offering informal caregivers a tailor-made program during rehabilitation and good communication helps to diminish caregiver burden in the post-discharge phase when the relative is back home.

## Figures and Tables

**Table 1:** Code list.

Code List
Communication
Future
Worrying
Social network
Energy level
Discharge
Knowledge
Skills
Walk-in days
Weekend leave
Informal caregiver meetings
Psychoeducation

**Table 2:** Participants and the interventions followed.

Participant	Gender	Age	Relation to Patient	Age Patient	Gender Patient	Recurrent Stroke	Formerly Informal Caregiver	KATZ Patient *	Informal Caregiver Meetings (1)	Walk-In Days (2)	Weekend Leave (3)	Psycho-Education (4)
1	Female	69	Spouse	68	Male	+	+	3	+	+	+	−
4	Female	78	Spouse	78	Male	−	−	6	+	+	+	−
8	Female	70	Spouse	70	Male	−	−	6	−	−	+	+
9	Female	43	Daughter	70	Male	−	−	6	−	−	+	−
10	Female	32	Daughter	69	Male	−	−	6	+	+	−	+
11	Male	31	Son	81	Male	−	+	4	+	+	−	−
13	Male	73	Spouse	72	Female	−	−	5	+	+	+	+
14	Female	70	Spouse	70	Male	−	−	4	+	+	+	−
15	Female	69	Spouse	69	Male	−	−	6	+	+	+	−
16	Female	55	Daughter	88	Female	+	+	0	−	−	−	−
17	Female	78	Spouse	82	Male	−	−	6	+	+	+	−

* completed by the informal caregiver in the first two weeks of admission; KATZ score from (0) low independence to (6) high independence.

## Data Availability

Due to privacy and ethical restrictions data is available on request to the corresponding author.
